# Evaluating the Potential Antibiotic Resistance Status in Environment Based on the Trait of Microbial Community

**DOI:** 10.3389/fmicb.2020.575707

**Published:** 2020-10-06

**Authors:** Zhiguo Su, Bei Huang, Qinglin Mu, Donghui Wen

**Affiliations:** ^1^College of Environmental Sciences and Engineering, Peking University, Beijing, China; ^2^Zhejiang Provincial Zhoushan Marine Ecological Environmental Monitoring Station, Zhoushan, China

**Keywords:** antibiotic resistance genes, microbial community, coastal sediment, predictive metagenomics, ribosomal RNA operon copy number

## Abstract

The overuse of antibiotics has promoted the propagation and dissemination of antibiotic resistance genes (ARGs) in environment. Due to the dense human population and intensive activities in coastal areas, the health risk of ARGs in coastal environment is becoming a severe problem. To date, there still lacks of a quantitative method to assess properly the gross antibiotic resistance at microbial community level. Here, we collected sediment samples from Hangzhou Bay (HB), Taizhou Bay (TB), and Xiangshan Bay (XB) of the East China Sea for community-level ARGs analysis. Based on the 16S rRNA genes and predictive metagenomics, we predicted the composition of intrinsic ARGs (piARGs) and some related functional groups. Firstly, a total of 40 piARG subtypes, belonging to nine drug classes and five resistance mechanisms, were obtained, among which the piARGs encoding multidrug efflux pumps were the most dominant in the three bays. Secondly, XB had higher relative abundances of piARGs and pathogens than the other two bays, which posed higher potential health risk and implied the heavier impact of long-term maricultural activities in this bay. Thirdly, the co-occurrence network analysis identified that there were more connections between piARGs and some potential pathogenic bacteria. Several piARG subtypes (e.g., *tetA*, *aacA*, *aacC*, and *aadK*) distributed widely in the microbial communities. And finally, the microbial diversity correlated negatively with the relative abundance of piARGs. Oil, salinity, and arsenic had significant effects on the variations of piARGs and potential pathogenic bacteria. The abundance-weighted average ribosomal RNA operon (*rrn*) copy number of microbial communities could be regarded as an indicator to evaluate the antibiotic resistance status. In conclusion, this study provides a new insight on how to evaluate antibiotic resistance status and their potential risk in environment based on a quantitative analysis of microbial communities.

## Introduction

In the coastal environment all over the world, the emergence of antibiotics and the spread of antibiotic resistance genes (ARGs) is believed to be promoted under anthropogenic impacts, such as wastewater disposal and mariculture (Niu et al., 2016; Zhang et al., 2018; Lu et al., 2020). In China, for example, high concentrations of antibiotic residues and ARGs are frequently detected in the seawater and sediments along the coastline (Zhang et al., 2013; Chen et al., 2017; Zhu et al., 2017; Chen J. et al., 2019; Li et al., 2020). There is an important fact that ARGs will persist even though antibiotic use is strictly restricted or even banned (Vaz-Moreira et al., 2014). In addition, many other pollutants (aromatics, heavy metals, and nutrients, etc.) discharged by human activities could favor the accumulation of ARGs in the coastal environment (Guo et al., 2016; Bengtsson-Palme et al., 2017; Wang et al., 2017). As sediment harbors a large number of various microbes, the diversity and abundance of ARGs in sediment could provide us a high-resolution vision to evaluate the long-term anthropogenic impacts.

Currently, the studies on ARGs in various environments mostly depend on polymerase chain reaction (PCR) and real-time quantitative PCR (qPCR; Jia et al., 2017; Li et al., 2018; Chen J. et al., 2019). But it is nearly impossible to profile the gross ARGs of an environmental sample due to the limited number of validated primers. Many previous studies suggested that the compositions of microbial community were strongly correlated with the diversity and abundance of ARGs in the environments (Su et al., 2015; Fan et al., 2018). Multiple intrinsic ARGs also represent native resistance form in the bacterial genomes and contribute an important fraction of environmental antibiotic resistome (Vaz-Moreira et al., 2014). On the other hand, the potential transmission of environmental ARGs to human pathogen also poses a great risk to human health (Ju et al., 2016; Fang et al., 2018). Therefore, in-depth analysis of microbial community compositions might be another route to evaluate the potential antibiotic resistance status and its risk. By now, there still lacks of a proper quantitative evaluation method at the level of microbial community.

In this study, we selected three typical bays of Zhejiang Province in China as our studied area. Based on high-throughput sequencing of 16S rRNA gene and predictive metagenomic methods (Tax4Fun, FAPROTAX, and BugBase), we comprehensively investigated the compositions of microbial communities and predicted the intrinsic ARGs (piARGs) in the coastal sediments. We aimed to (1) explore the microbial resistance status under long-term stress of human activities in the coastal environment; (2) elucidate the impacts of microbial quality and environmental factors on the emergence of piARGs; and (3) identify the internal relationship between antibiotic resistance status and key traits of microbial community. This study will provide a quantitative method and potential indicator to evaluate the antibiotic resistance status and risk of coastal environment at the microbial community level.

## Materials and Methods

### Study Area and Samples Collection

Twenty sampling sites in Hangzhou Bay (HB), Taizhou Bay (TB), and Xiangshan Bay (XB) were chosen, as shown in Supplementary Figure S1. The three bays locate in the coastal area of the East China Sea, which was suffering from severe deterioration of ecosystem (MEE, 2019). According to our previous studies, HB and TB are surrounded by many cities, industrial parks, and pharmaceutical bases in Zhejiang Province (Su et al., 2018; Chen J. et al., 2019). Their ecological status is influenced by lots of human activities such as wastewater discharge, coastal engineering, industrial, and agricultural activities. XB, an important maricultural base in Zhejiang Province, is subject to a large influx of antibiotics from the rapid development of aquaculture (Gao et al., 2012; Xiong et al., 2015). In addition, the weak wave might lead to high accumulation of antibiotics in this semi-enclosed narrow bay (Li et al., 2017a; Chen et al., 2018). Many other pollutants are also frequently detected in the three bays, including inorganic nitrogen, phosphate, organic pollutants, and heavy metals (Chen J. et al., 2019).

The sediment samples at 20 sites were collected in April 2017. At each site, we collected triplicate surface sediment (0–5 cm) samples within a 10 m × 10 m area by using a grab sampler (Van Veen, Hydro-Bios, Germany) and overlying seawater samples by using organic glass deep-water samplers. All sediment samples (*n* = 60) were sealed in airtight sterile plastic bags and stored at −70°C after transportation to our laboratory. The physicochemical properties of all the sediment (i.e., oil, heavy metals, total phosphorus, total nitrogen, and total organic carbon) and overlying seawater (i.e., pH, salinity, and temperature) samples were determined by standard methods (GB18668-2002). The results are shown in Supplementary Table S1.

### DNA Extraction, PCR, and 16S rRNA Gene Sequencing

The total microbial DNA was extracted from each 0.5 g lyophilized sediment sample using a Power Soil DNA Isolation Kit (Mo Bio, United States), according to the manufacturer’s protocol. The concentration and purity of an extracted DNA sample were determined using a NanoDrop ND-2000 Spectrophotometer (Thermo Scientific, United States). The V4 region of the 16S rRNA gene was amplified by PCR with specific primers 515F and 806R (Caporaso et al., 2011). The PCR amplification program and amplicons sequencing followed the method in our previous studies (Zheng et al., 2019). Raw sequence data were deposited into the NCBI Sequence Read Archive (SRA) database (PRJNA414257).

### Processing of Sequencing Data

Raw reads were filtered in order to eliminate the low quality and ambiguous reads using the Trimmomatic (V0.33^1^). Paired-end clean reads were merged using FLASH (V 1.2.11^2^). After quality control, the pick_open_reference_otus.py workflow on the QIIME (V 1.9.1^3^) platform was used to pick operational taxonomic units (OTUs) with 97% sequence similarity within the reads and pick a representative sequence from each OTU. RDP classifier in QIIME pipeline was used to assign taxonomic data to each representative sequence against the SILVA (Release 132^4^) 16S rRNA gene database. To equalize the sampling efforts across samples, based on the lowest sequencing depths, the subset of 49,069 sequences from each sample was randomly selected for further analysis. The number of filtered sequences and normalized number of observed OTUs are shown in Supplementary Table S2. Checking on the OTUs data, TC0b was abnormal and very different from TC0a and TC0c. We kept the data of 59 samples except TC0b for subsequent analysis.

### Bioinformatics Analysis for Predictive Function

Tax4Fun,^5^ an R package was applied to predict the functional capabilities of microbial communities according to the 16S rRNA gene sequence data (Aßhauer et al., 2015). The output of Tax4fun consisted of a table of functional gene counts known as KEGG Orthologs (KOs). The KOs related to antibiotic resistance were obtained by manually screening the subtypes of ARGs provided in KEGG database.^6^ These genes represent a part of intrinsic ARGs stored in the microbial community. And drug classes and resistance mechanisms of these ARGs were classified based on the CARD database.^7^

FAPROTAX^8^ determines the microbial metabolic functions depending strictly on the known information of culturable species (Louca et al., 2016). In this study, 84 functional groups were represented and 15.6% of the total OTUs were assigned to at least one group based on FAPROTAX workflow. Then we used BugBase,^9^ an algorithm to predict organism-level coverage of functional pathways as well as biologically interpretable phenotypes, such as pathogenic potential and mobile elements containing (Ward et al., 2017). The estimation of ribosomal RNA operon (*rrn*) copy number for each OTU was conducted based on *rrn*DB database^10^ (Klappenbach et al., 2001). Then the abundance-weighted average *rrn* copy number of each community was calculated according to a previous study (Wu et al., 2017). In addition, the characterization and quantification of ARGs in the environmental metagenomes were solved using ARGs-OAP v2.0 (Yin et al., 2018).

### Statistical Analysis

Rarefaction curve (Supplementary Figure S2) and alpha diversity indexes (Supplementary Table S2) were calculated for each sample using QIIME. Significant differences between different bays for piARGs and other biotic indexes were evaluated using Wilcoxon test. Linear regression model was used to determine the relationships between piARGs and some biotic indexes or microbial diversity. Redundancy analysis (RDA) and variance partition analysis (VPA) were employed to identify key factors affecting piARGs and potential pathogenic bacteria. Correlation analysis was used between *rrn* copy number and other microbial indexes related to tolerance and virulence. These statistical analyses were performed in vegan, ggpubr, PerformanceAnalytics, and ggplot2 package of R software (v.3.6.0).

Co-occurrence networks were constructed to identify the connection between piARGs and microbial genus or function, respectively. At the beginning of network analysis, the Spearman’s correlation matrix in the different bays was calculated. All the statistically robust correlations (*p* < 0.01 and Spearman’s *r* > 0.8 for microbial genus; *p* < 0.01 and Spearman’s *r* > 0.7 for microbial function) formed the co-occurrence networks. These statistical analyses were performed using vegan, psych, and igraph packages in R software (v.3.6.0). Then, the network visualization and its parameter calculation were conducted on the Gephi (0.9.2).

## Results and Discussion

### Composition and Distribution of piARGs in the Three Bays

**Figure 1:**
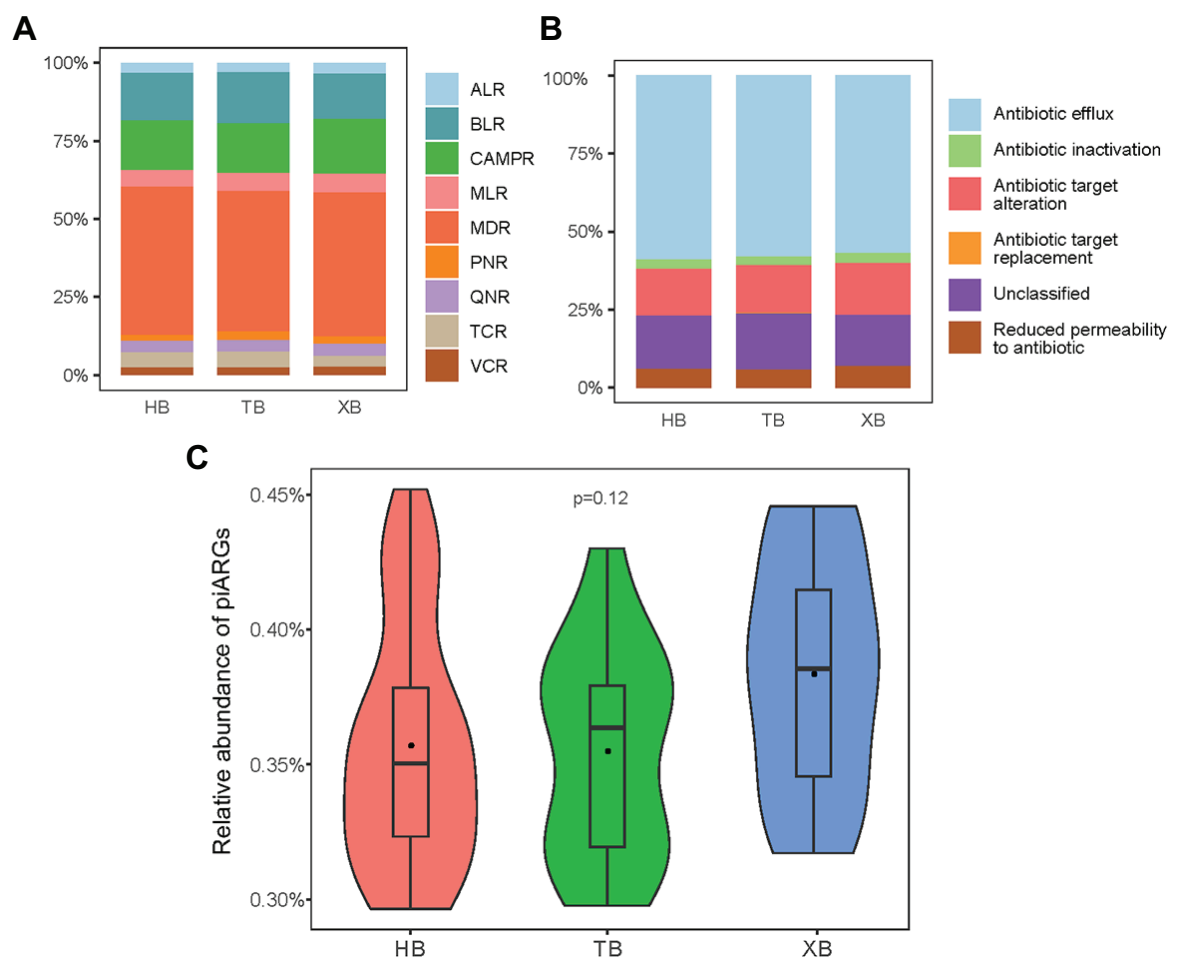
Predicted antibiotic class **(A)** and resistance mechanism **(B)** of predicted the intrinsic antibiotic resistance genes (piARGs). The relative proportion of total piARGs in the three bays **(C)**. ALR, aminoglycoside resistance genes; BLR, beta-lactam resistance genes; CAMPR, cationic antimicrobial peptide resistance genes; MLR, macrolide resistance genes; MDR, multidrug resistance genes; PNR, phenicol resistance genes; QNR, quinolone resistance genes; TCR, tetracycline resistance genes; VCR, vancomycin resistance genes.

In our study, the observed OTUs and Good’s coverage values indicated that the constructed libraries could well reflect the samples’ microbial communities (Supplementary Table S2). And the rarefaction curves generated from the OTUs also suggested satisfactory coverage in all samples (Supplementary Figure S2). The occurrence of piARGs in the three bays was investigated. In total, 40 predicted ARG subtypes potentially conferred resistance to nine classes of antibiotics, including aminoglycoside (8), macrolide (5), beta lactam (7), vancomycin (3), cationic antimicrobial peptide (8), tetracycline (2), phenicol (1), quinolone (1), and multidrug (5; Supplementary Table S3). As Figure 1A shown, the relative proportion of piARGs conferring resistance to multidrug antibiotics (MDR, 46.6%) was dominant in the three bays. Beta-lactam resistance genes (BLR, 15.4%) and cationic antimicrobial peptide resistance genes (CAMPR, 16.4%) were also main gene types. On the other hand, most of the predicted ARGs belonged to five resistance mechanisms (antibiotic inactivation, antibiotic target alteration, antibiotic efflux pump, antibiotic target replacement, and reduced permeability to antibiotic); and some other genes were unclassified. Antibiotic efflux pump (58.3%) was the most important resistance mechanism of these piARGs, followed by unclassified (16.4%) and antibiotic target alteration (14.6%) mechanism (Figure 1B). Above findings were consistent with previous studies. Multidrug efflux pump was reported to be one of the most common resistance mechanisms in the environment such as estuary and deep ocean sediments (Du et al., 2018). In addition, ARGs encoding multiple antibiotic resistance were frequently found in polluted areas, indicating human activities might be important driving force on the development of MDR (Gao et al., 2012; Wang et al., 2018).

As for the total relative abundance of piARGs in the three bays, XB was higher than the other two bays, but the difference was insignificant based on anova (Figure 1C). This result indicated that XB might be under more serious ARGs pollution due to the long-term impact of maricultural activities in XB. A previous study also revealed that the abundance of ARGs in the maricultural sediments of China’s coastline (including XB) was higher than a non-aquaculture site (Gao et al., 2018). Overall, compared with the traditional qPCR method, predictive metagenomics enable us to profiled a more comprehensive ARG status quickly. Meanwhile, since the intrinsic resistance genes were related to microbes directly, piARGs might be more suitable to represent long-term anthropogenic impacts on the variation of antibiotic resistance status.

### Microbial Quality and Antibiotic Resistance in the Three Bays

**Figure 2:**
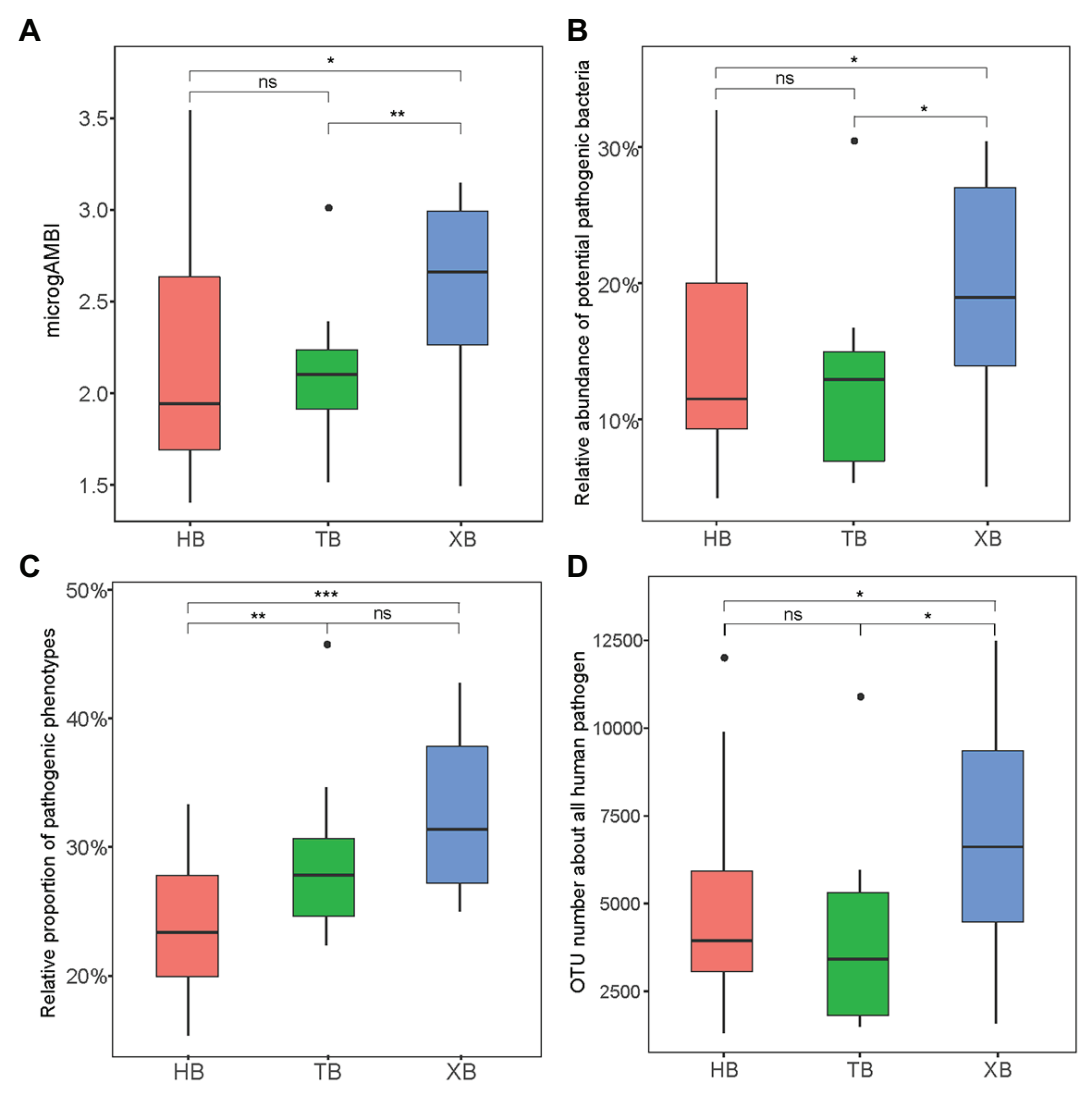
The microbial indexes of tolerance and virulence in the three bays. microgAMBI was calculated according to previous research **(A)**; Potential pathogenic bacteria was classified based on PHI-base (http://www.phi-base.org/; **B**); Pathogenic phenotype was predicted by BugBase (https://bugbase.cs.umn.edu/; **C**); operational taxonomic unit (OTU) number of all human pathogen was predicted by FAPROTAX **(D)**. The Wilcoxon test was applied in comparing the intergroup difference (^*^ *p* < 0.05, ^**^p < 0.01, ^***^p < 0.001, ns means insignificant difference).

The microbial quality of environment is significantly related to various contamination sources, so far mainly focusing on the distribution of fecal indicator bacteria or human pathogens (Cui et al., 2017). These specific bacterial compositions have been regarded as of great concern to ecological and human health. Here, microgAMBI is a new biotic index based on the bacterial assemblage composition at family level for the ecological status assessment of coastal environment (Aylagas et al., 2017). According to the proposed ecological quality classes indicated by microgAMBI (high, good, moderate, poor, and bad), the environmental status of the three bays were not “poor” nor “bad” (microgAMBI ≤ 3.6). But the microgAMBI values of XB were higher than those of HB and TB significantly (Figure 2A), further indicating that XB might be under higher anthropogenic pressures.

Moreover, potential pathogens could confer antibiotic resistance and pathogenicity, posing a great risk to public health (Fan et al., 2018). Therefore, as an important index the composition and abundance of potential pathogens should be paid more attention in the evaluation of antibiotic resistance status. In this study, we classified and predicted the contribution of potential pathogens in the microbial community from different angles. Firstly, the reference database of human pathogenic bacteria (Pathogen Host Interaction, PHI-base) was used to identify potential pathogens in the sequencing data. In total, there were 39 genera identified in the three bays. As Supplementary Figure S3 showed, *Acinetobacter* and *Citrobacter* were the most abundant potential pathogenic bacteria, which were reported frequently in polluted areas (Xiong et al., 2015). And the total relative abundance of potential pathogenic bacteria was significantly higher in XB than the other two bays (Figure 2B). Secondly, based on predictive metagenomics methods (BugBase and FAPROTAX), we predicted the relative proportion of pathogenic phenotypes (Figure 2C) and the OTU number of human pathogens (Figure 2D) in our studied area. These results also showed significant difference of pathogenic composition among the three bays. XB had higher proportion of potential pathogens, frequently containing genes for virulence factors (Liang et al., 2020). Of course, the identification of pathogens from 16S rRNA gene sequence mainly depends on limited reference database in this study.

**Figure 3:**
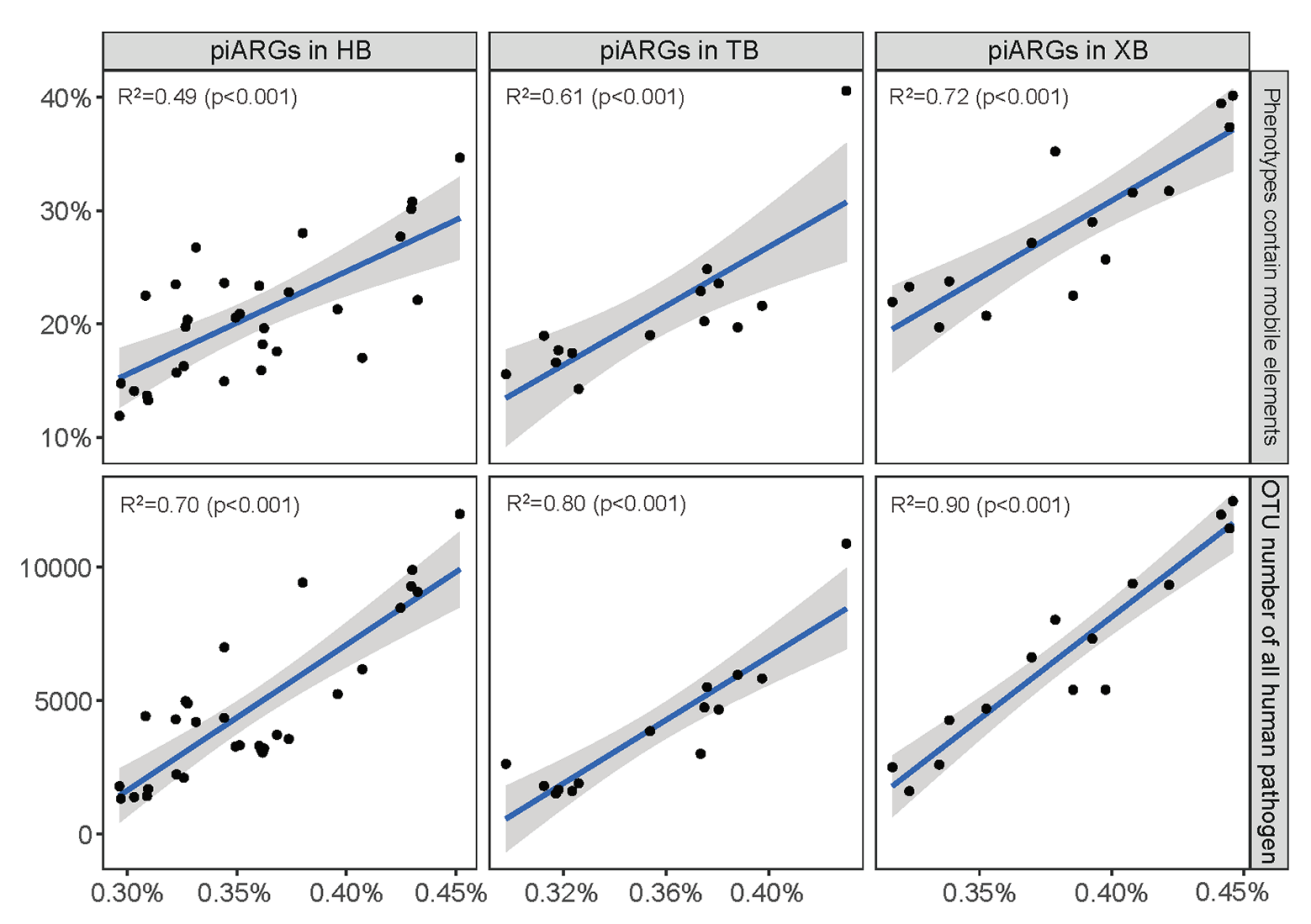
Linear regression model showed the relationships between piARG abundance and proportion of phenotypes containing mobile elements, OTU number of all human pathogen in the three bays. The solid lines indicate significant relationships. The shaded areas show the 95% confidence interval of the fit.

Furthermore, linear regression model was used to determine the relationships between piARG and mobile elements, human pathogen in the three bays, respectively. As Figure 3 showed, the proportion of predicted phenotypes containing mobile elements showed a positive correlation with the relative abundance of piARGs. And the increase in OTU number of human pathogens was linearly associated with an increase in piARG abundance. These correlations in XB (*R*^2^ = 0.72 for mobile elements and *R*^2^ = 0.90 for human pathogen) were stronger than those in HB (*R*^2^ = 0.49 and *R*^2^ = 0.70) and TB (*R*^2^ = 0.61 and *R*^2^ = 0.80). According to the previous studies, mobile genetic elements (MGEs) played pivotal roles in modulating not only the horizontal transfer of ARGs but also the adaptation of microbial community to pollutants (Top and Springael, 2003; Holmes et al., 2016). Therefore, these results showed higher transfer frequency of piARGs in the microbial community of XB, likely indicating the microbial adaptation mechanism to the local pollution. The potential pathogens contributed more antibiotic resistance in the microbial community of XB, posing a higher risk to ecological environment and human health (Ju et al., 2016; Fan et al., 2018). In a word, the analysis of microbial quality is beneficial to evaluate ecological health status and potential antibiotic resistance risk in the environment.

### Co-occurrence Network Analysis Between piARGs and Microbial Community

Network analysis was applied to explore the relationships among piARGs, common microbial genera (detected in more than 80% of samples) and some fundamental functional groups (i.e., cycles of carbon, nitrogen, sulfur, and degradation metabolism, shown in Supplementary Table S4) in the three bays.

**Figure 4:**
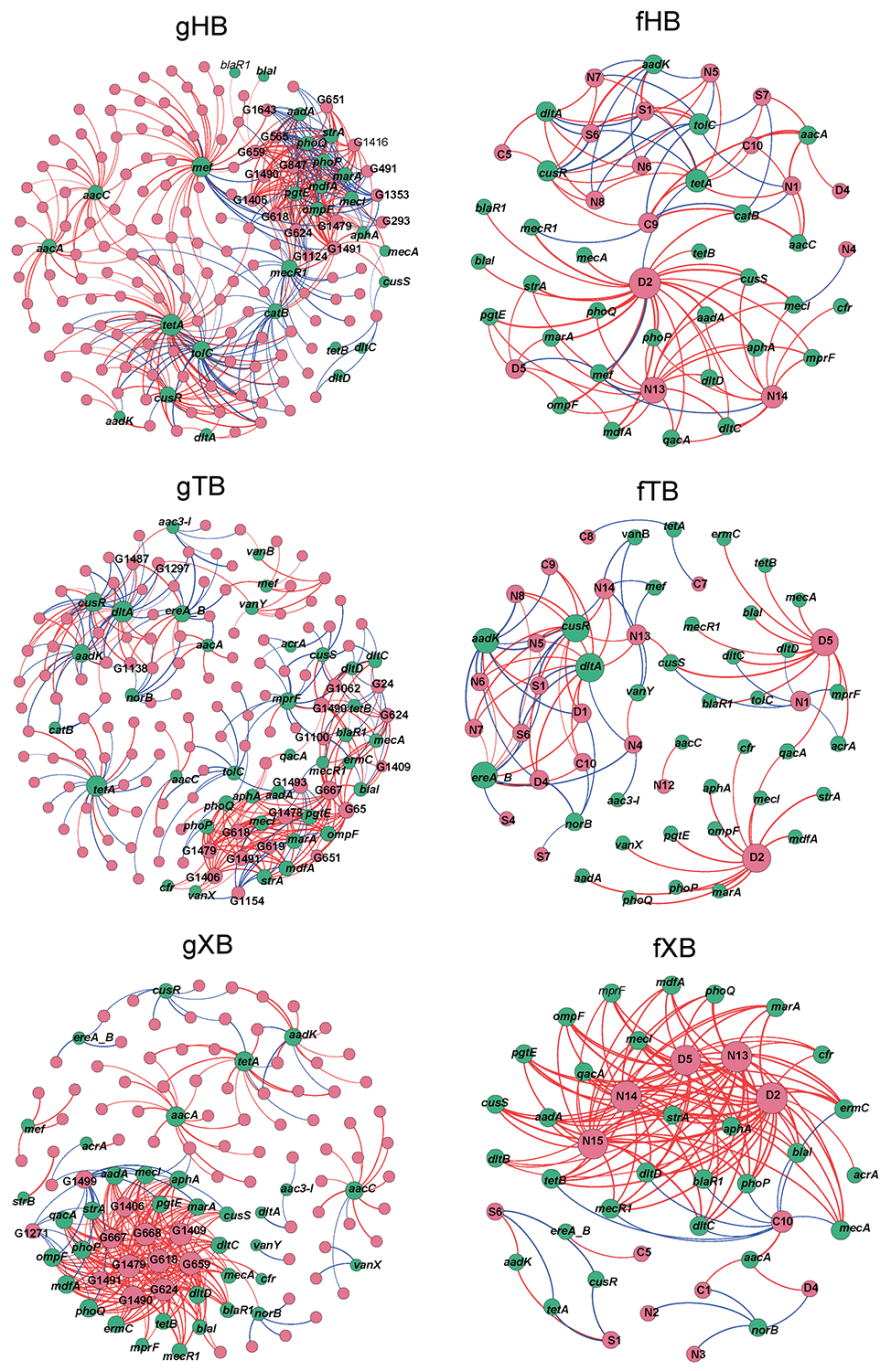
Co-occurrence network analysis of piARGs and common microbial genus (gHB, gTB, and gXB), functional groups (fHB, fTB, and fXB) in the different bays. gHB and fHB showed networks for HB, gTB, and fTB showed networks for TB, gXB, and fXB showed networks for XB. A node stands for a piARG subtype (green), a genus, or a functional group (pink). The node size is proportional to the number of connections (degree) and the edge thickness is proportional to the value of Spearman’s r. Edge colors are used to distinguish positive (red) or negative (blue) correlation.

As Figure 4 showed, the gHB, gTB, and gXB described the co-occurrence of piARGs and common microbial genera in the three bays, respectively. The total nodes (160) and edges (406) of gHB were higher than the other two networks, indicating the higher complexity of gHB (Supplementary Table S5). Some key microbial genera and piARGs were identified, which had more connections with other nodes (node degree > average degree of network) in the networks. As for microbial genera, most of the nodes with higher degree in the network were identified as potential pathogenic bacteria such as *Acinetobacter*, *Enterococcus*, *Lactococcus*, *Citrobacter*, *Pseudomonas*, *Bacillus*, *Klebsiella*, and *Streptococcus* (Supplementary Table S6). Especially in the gXB, these potential pathogenic genera related to more piARGs. Previous studies also consistently reported the presence of some potential pathogenic genera in aquaculture system and their important roles in antibiotic resistance spread (Vaz-Moreira et al., 2014; Xiong et al., 2015). In this study, most of the highly connected genera belonged to Gammaproteobacteria and Bacilli, which were predominant bacterial classes in the sediments of disturbed coastal environment (Lu et al., 2016; Su et al., 2018; Supplementary Figure S4). It suggested that abundant and tolerant species harbored more piARGs under the effects of human activities. Based on the analysis of bacterial genomes, Proteobacteria and Firmicutes species were also highly enriched with many ARGs (Hu et al., 2016). In addition, the stronger connectivity between potential pathogenic genera and piARGs in the gXB indicated that pathogens tended to acquire ARGs likely due to their exposure to higher concentrations of antibiotics (Liang et al., 2020). The networks also indicated that some piARG subtypes (*tetA*, *aacA*, *aacC*, *aadK*, *cusR*, etc.) distributed widely in the microbial communities. Interestingly, based on qPCR detection, *tetA* was determined to be the most abundant ARG in XB (Gao et al., 2018), and tetracycline resistance genes were also previously proved to be prevalent in the aquaculture environment (Gao et al., 2012). Yang et al. (2013) also revealed that most of metagenomic sequences encoded aminoglycoside and tetracycline resistance in the marine sediment samples.

The fHB, fTB, and fXB described the co-occurrence of piARGs and some functional groups in the three bays, respectively. In these networks, two functional groups related to aromatic compound degradation and plastic degradation had most connections with piARGs (Supplementary Table S4). This result indicated that piARGs were mostly relevant with degradation and/or metabolism of refractory contaminants. Previous studies found that PAHs could accelerate the propagation of ARGs in coastal microbial community (Wang et al., 2017). Many PAH catabolic genes resided in the MGEs (Koenig et al., 2009), indicating the effect of horizontal transfer on the co-occurrence of piARGs and aromatic compound degradation genes (ADGs). In addition, there were 67.6% of the public available bacterial genomes harboring ARGs and ADGs (Xia et al., 2019). As for the strong connection between piARGs and plastic degradation genes, it might be related to the impact of more and more microplastics on the transmission of ARGs in the environment (Arias-Andres et al., 2018). The functional groups about nitrate reduction process (nitrate respiration, nitrate reduction, and nitrogen respiration) also had more significant correlations with piARGs, especially in the fXB. It was reported that many different types of antibiotics played vital roles in the nitrate reduction process, especially denitrification (Hou et al., 2015; Yin et al., 2017). Meanwhile, horizontal gene transfer for both denitrification and antibiotic resistance had been observed to occur in the microbial community (Sun et al., 2017). And some denitrifiers likely acquired ARGs to maintain their denitrifying functions under the exogenous stress (Sun et al., 2017). Our results also proved these findings and indicated that microbial antibiotic resistance could play an important role in the co-regulation of nitrate and antibiotics metabolism in the environment. As the primary pollutants in the studied area, the impact of nitrate on the emergence of ARGs should be paid more attentions. In addition, as Supplementary Table S5 showed, most of the positive correlations were shown in gXB (82.23%) and fXB (87.59%). It also suggested that piARGs in XB showed stronger connectivity with tolerant genera and functions and XB, influenced by mariculture, might have higher potential antibiotic resistance risk. Network analysis revealed the deep relationship between piARGs and microbial community, while provided comprehensive sights of the effects of antibiotic resistance on the ecological process.

### Roles of Microbial Diversity and Environmental Factors on the Variation of piARGs

**Figure 5:**
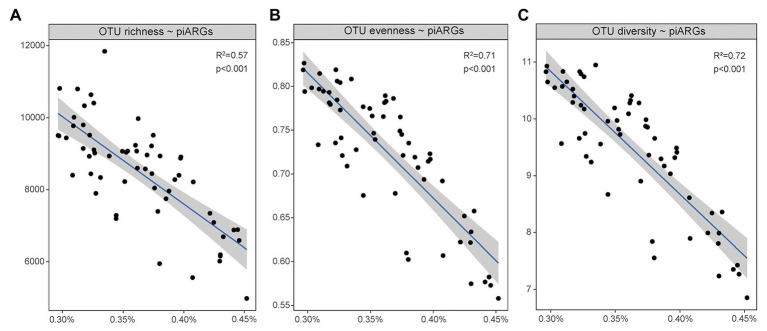
Linear regression model showed the relationships of piARG abundance with OTU richness **(A)**, OTU evenness **(B)**, and OTU diversity **(C)**.

As Figure 5 showed, a linear regression model was used to determine the relationship between microbial diversity (Supplementary Table S2) and the relative abundance of piARGs. It is well known that microbial diversity played an important role in the resistance to microbial invasion in the natural ecosystem (van Elsas et al., 2012). Some studies regarded the antibiotic resistome as a proxy for invasion and found that microbial diversity had negative correlation with the abundance of ARGs (Chen Q. et al., 2019). In this study, an increase in piARG abundance was linearly associated with loss of OTU richness (observed OTUs, *R*^2^ = 0.57, *p* < 0.001), OTU evenness (Pielou index, *R*^2^ = 0.71, *p* < 0.001), and OTU diversity (Shannon index, *R*^2^ = 0.72, *p* < 0.001). So, our results also indicated that microbial diversity could construct a biological barrier to prevent the invasion of antibiotic resistance in the coastal ecosystem. But lower microbial diversity was usually observed in higher disturbed environments, indicating the potential higher frequency of antibiotic resistance development.

**Figure 6:**
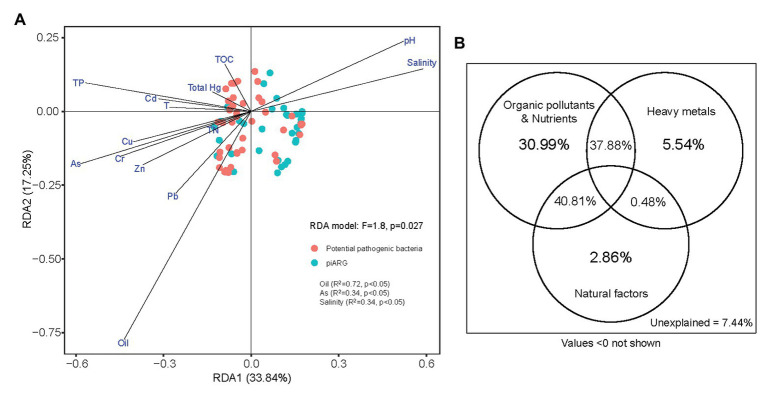
Redundancy analysis of piARGs, potential pathogenic bacteria, and environmental factors **(A)**; and variation portioning analysis (VPA) differentiating the contributions of heavy metals, organic pollutants, and natural factors on the variation of piARGs and potential pathogenic bacteria **(B)**.

Moreover, the dissemination of antibiotic resistance in environments was associated with other resistance determinants such as heavy metals, biocides, or organic pollutants (Vaz-Moreira et al., 2014). In this study, we determined 11 factors (oil, seven different heavy metals, total phosphorus, total nitrogen, and total organic carbon) for sediment and three factors (pH, salinity, and temperature) for overlying seawater, in order to represent the basic physicochemical properties of collected samples (Supplementary Table S1). RDA model (*F* = 1.8, *p* = 0.027) indicated that oil (*R*^2^ = 0.72, *p* < 0.05), arsenic (*R*^2^ = 0.34, *p* < 0.05), and salinity (*R*^2^ = 0.34, *p* < 0.05) played key roles in the compositions of piARGs and potential pathogenic bacteria (Figure 6A). In our previous study, oil was also identified as the most important factors influencing the distribution of ARGs (Chen J. et al., 2019). It also supported the relationship between piARGs and functional group of aromatic compound degradation. Many studies had proved that co-resistance is an important mechanism of antibiotic-metal co-selection (Li et al., 2017b). Arsenic, as an important heavy metal in this study, shared several resistance mechanisms (e.g., reduction in permeability, target alteration, and efflux pumps) with many types of antibiotics including tetracycline, β-lactams, chloramphenicol, and ciprofloxacin (Baker-Austin et al., 2006). In addition, salinity, as a key natural factor regulating the distributions of ARGs and pathogens in the coastal environments, also was proved in previous studies (Bergeron et al., 2016; Zhang et al., 2019). As Figure 6B shown, VPA further identified that organic pollutants and nutrients (i.e., oil, total organic carbon, total phosphorus, and total nitrogen) contributed more (30.99%) to the variation of piARGs and potential pathogenic bacteria in the three bays. The joint effects (40.81%) of organic pollutants and nutrients and natural factors (i.e., temperature, pH, and salinity) were crucial. It also indicated that environmental nutrients and organic pollutants could promote the transmission of ARGs in the microbial community (Guo et al., 2015). Except for salinity, temperature or pH also played key roles in the variation of ARG abundance (Verma et al., 2002). Overall, environmental resistome could be influenced by various biotic (microbial diversity) and abiotic factors (physicochemical parameters) in the coastal area. Therefore, it is necessary to understand the complex effect of environment on the spread of ARGs in order to control the potential risk.

### *rrn* Copy Number of Microbial Communities as an Indicator to Evaluate the Antibiotic Resistance Status

**Figure 7:**
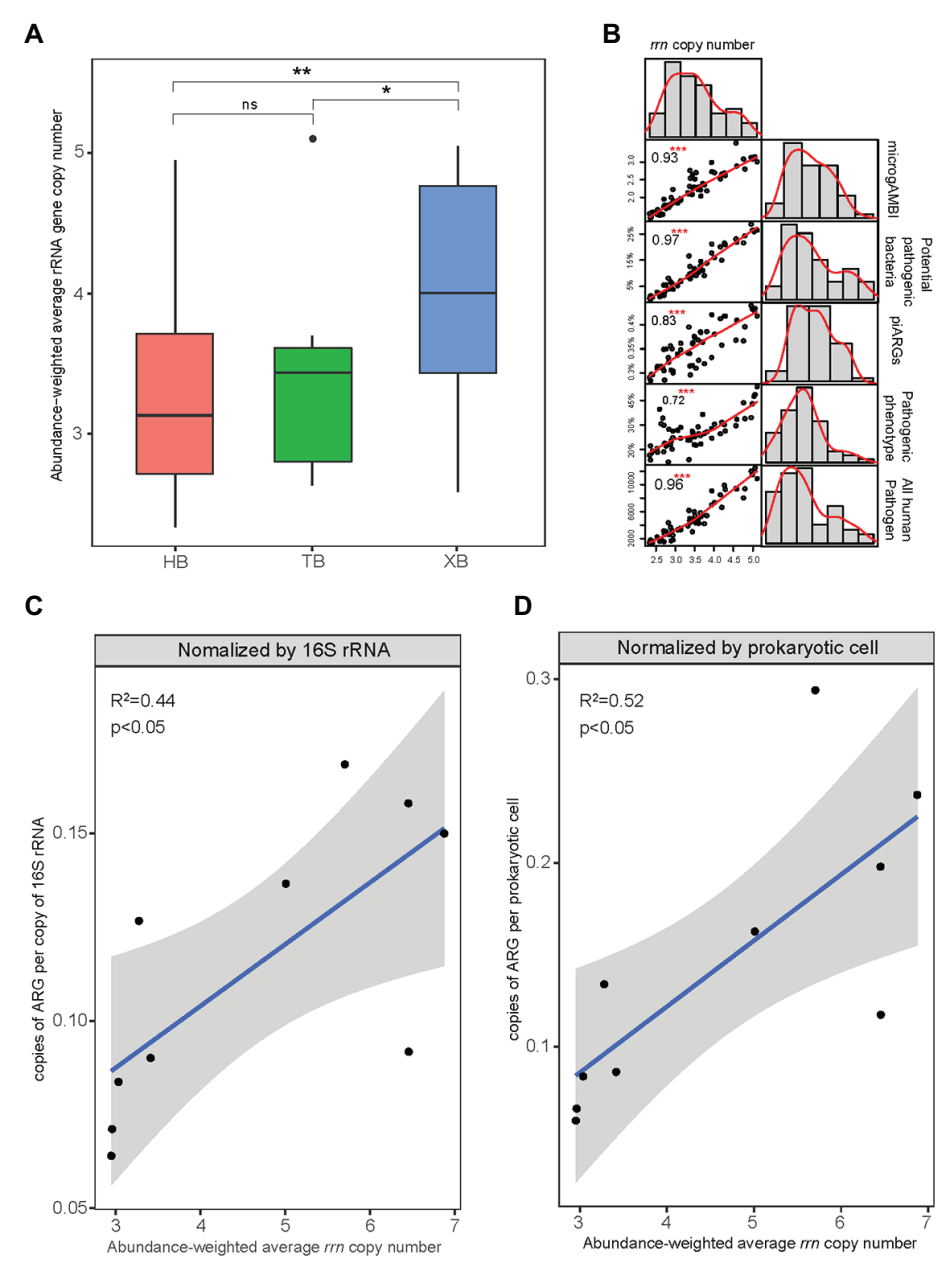
Abundance-weighted average rRNA gene copy number of microbial communities in the three bays (^*^p < 0.05, ^**^p < 0.01, ns means insignificant difference; **A**) and their correlation with other microbial indicators of tolerance and virulence (^***^p < 0.001; **B**). The relationship between abundance-weighted average ribosomal RNA operon (*rrn*) copy number and copies of ARGs (metagenomics data) according to previous studies **(C,D)**.

The abundance-weighted average *rrn* copy numbers for all samples of the three bays were calculated and shown in Figure 7A. The abundance-weighted average *rrn* copy numbers of microbial community in XB were significantly higher than those in HB and TB. This result was consistent with the comparison of other microbial indexes related to tolerance and virulence in the three bays. In some studies, *rrn* copy number was regarded as a key trait to indicate the life strategy of microorganisms (Wu et al., 2017). The higher abundance-weighted average *rrn* copy numbers of microbial community represent the higher proportions of *r*-strategists, which have high growth rate but low resource use efficiency (Roller et al., 2016). In this study, as Figure 7B showed, the abundance-weighted average *rrn* copy number had strong and positive correlations with piARGs and other microbial indexes related to tolerance and virulence. It suggested that *r*-strategists could be more likely to acquire ARGs in the microbial communities and their high growth rate would promote the propagation of ARGs.

Furthermore, we determined the linear relationship between copies of ARGs (Supplementary Tables S7 and S8) and *rrn* copy number (Supplementary Table S9) based on the metagenomics data of HB from previous studies (Figures 7C,D; Dai et al., 2016, 2018). And the copies of ARGs normalized by prokaryotic cell showed higher correlation (*R* ^2^ = 0.52, *p* < 0.05) with *rrn* copy number, indicating that *rrn* copy number was more likely to represent the intrinsic antibiotic resistance. Moreover, *rrn* copy number strongly associated with different physiological features of prokaryotes, including growth rate, transcription activation, molecular signaling, and motility (Dai et al., 2020). These features help microbes to adapt to exogenous stress and correlate with the development of cellular antibiotic resistance. Because ARGs transfer promoted by environmental pollutants exposure related significantly to various microbial cellular activities such as SOS response (Lu et al., 2018). It also explains why higher ARGs copies or more frequent gene transfer occurred in the several specific microbial taxa (Hu et al., 2016). As *rrn* copy number plays an important role in combining the variation of microbial compositions and the propagation of ARGs, our study came up with the suggestion that abundance-weighted average *rrn* copy number, as the fundamental trait of microbial community, be an indicator to evaluate the antibiotic resistance status in the coastal environment.

In this study, we aim to regard piARGs as a kind of microbial trait to reflect the antibiotic resistance status at the level of microbial community. So, these piARGs identified by predictive approaches might not represent the real composition of ARGs detected by qPCR and metagenomics. This study just provides a new insight on understanding the community risk of antibiotic resistance and selecting a microbial indicator. Of course, the accuracy and practicality of these predictive methods should be improved in the following studies. On the one hand, the updated predictive software could be used for obtaining more comprehensive functional profiles based on 16S rRNA gene sequencing. On the other hand, to conduct metagenomic sequencing and multivariate statistical analysis will also improve the accuracy of predictive results.

## Conclusion

In this study, based on predictive metagenomics, the in-depth analysis of microbial communities was conducted to explore the antibiotic resistance status in the coastal environment influenced by long-term human activities. Most of piARGs conferred resistance to multidrug and efflux pumps were dominant in the sediments of the three bays in the East China Sea. XB had worse antibiotic resistance status and higher potential risk, likely due to the overuse of antibiotics in the mariculture area. The relationships between the piARGs and the microbial compositions and predicted functions clearly pointed out that the variation of antibiotic resistance status could be represented by some key traits of microbial communities, such as pathogenic phenotypes and xenobiotic biodegradation. Abundance-weighted average *rrn* copy number could be regarded as an indicator to evaluate the potential antibiotic resistance status and risk. Our study provided a novel and quick method to profile the gross antibiotic resistance at the community level and to evaluate the potential risk in environments under anthropogenic impacts. And this method would be verified and improved with the development of sequencing and bioinformatic techniques.

## Data Availability Statement

The datasets presented in this study can be found in online repositories. The names of the repository/repositories and accession number(s) can be found below: https://www.ncbi.nlm.nih.gov/, PRJNA414257.

## Author Contributions

ZS and DW conceived this study. BH and QM performed the sampling. ZS performed original data analysis and drafted the manuscript. DW contributed to the final manuscript. All authors contributed to the article and approved the submitted version.

### Conflict of Interest

The authors declare that the research was conducted in the absence of any commercial or financial relationships that could be construed as a potential conflict of interest.

## Footnotes

^1^http://www.usadellab.org/cms/?page=trimmomatic

^2^https://ccb.jhu.edu/software/FLASH/

^3^http://www.qiime.org/

^4^https://www.arb-silva.de/

^5^http://tax4fun.gobics.de/

^6^https://www.kegg.jp/

^7^https://card.mcmaster.ca/home

^8^http://www.zoology.ubc.ca/louca/FAPROTAX

^9^https://bugbase.cs.umn.edu/

^10^https://rrndb.umms.med.umich.edu/

## Abbreviations

ARGs: Antibiotic resistance genes
piARGs: Predicted intrinsic antibiotic resistance genes
MGEs: Mobile genetic elements
rrn: Ribosomal RNA operon
ADGs: Aromatic compound degradation genes
PCR: Polymerase chain reaction
qPCR: Real-time quantitative polymerase chain reaction
OTU: Operational taxonomic unit
KOs: KEGG orthologs
RDA: Redundancy analysis
VPA: Variance partition analysis
ALR: Aminoglycoside resistance genes
BLR: Beta-lactam resistance genes
CAMPR: Cationic antimicrobial peptide resistance genes
MLR: Macrolide resistance genes
MDR: Multidrug resistance genes
PNR: Phenicol resistance genes
QNR: Quinolone resistance genes
TCR: Tetracycline resistance genes
VCR: Vancomycin resistance genes
HB: Hangzhou Bay
TB: Taizhou Bay
XB: Xiangshan Bay.
