# Supplementary material for: Evaluating the Potential Antibiotic Resistance Status in Environment Based on the Trait of Microbial Community
Source: Front Microbiol. 2020 Oct 6;11:575707. doi: 10.3389/fmicb.2020.575707 (PMC7573184; doi:10.3389/fmicb.2020.575707)
Supplement: Supplementary file 1 [file Data_Sheet_1.docx]

***Supplementary Material***

**
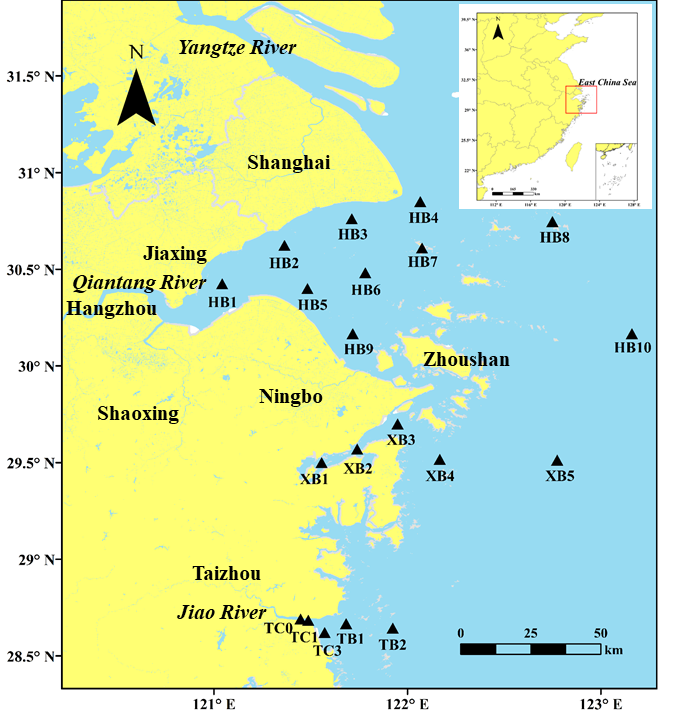
**

**Supplementary Figure 1**. The locations of sampling sites in the three bays in the East China Sea. HB1-HB10: the sampling sites in Hangzhou Bay (HB); XB1-XB5: the sampling sites in Xiangshan Bay (XB); TC0, TC1, TC3, TB1, and TB2: the sampling sites in Taizhou Bay (TB).


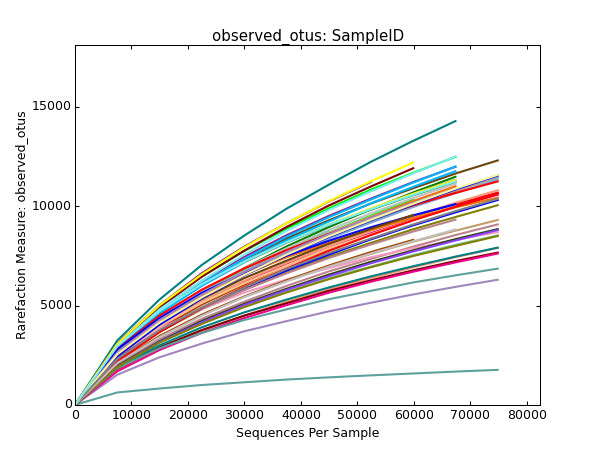


**Supplementary Figure 2**. The rarefaction curves of microbial communities.


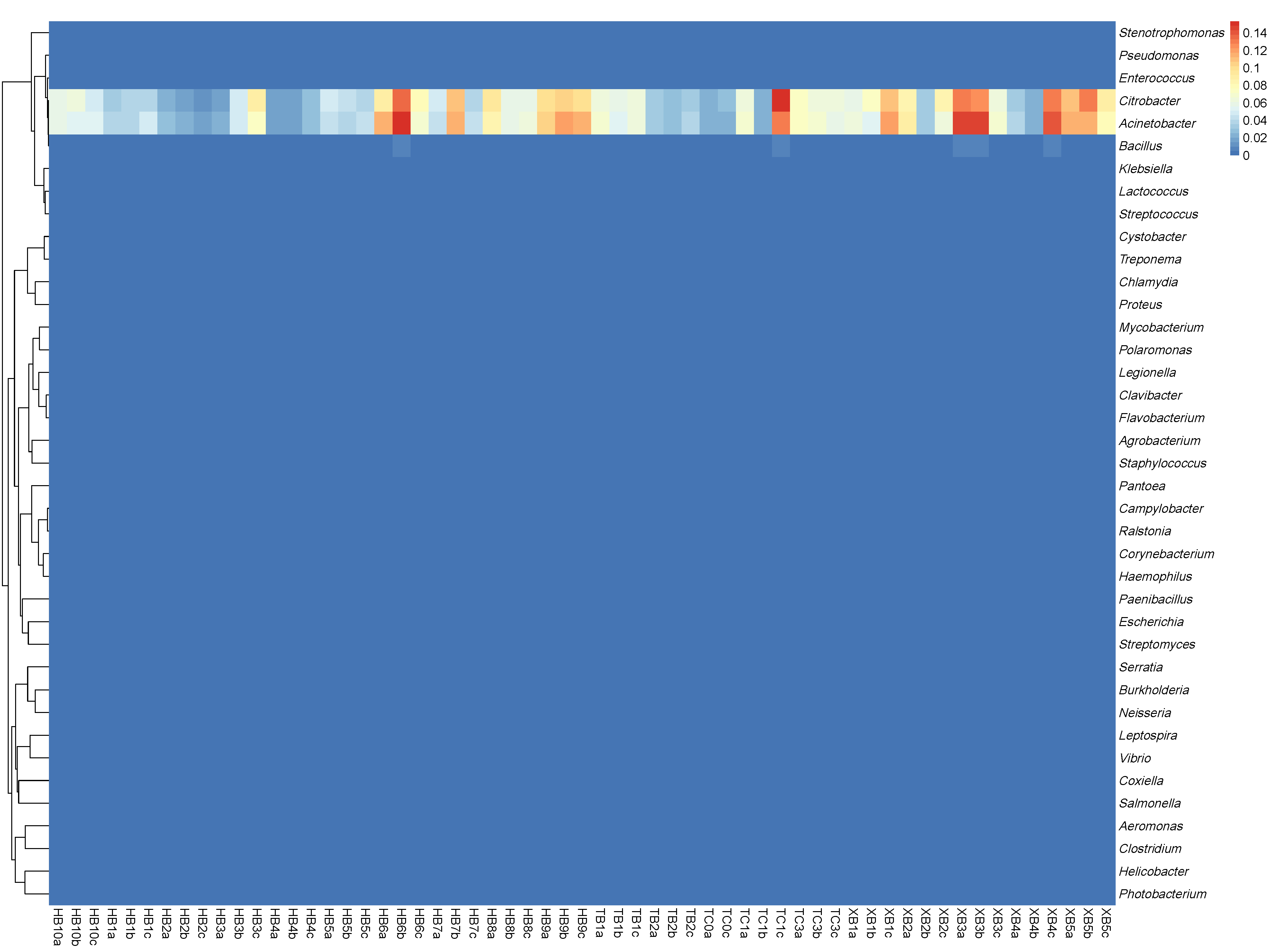


**Supplementary Figure 3.** Relative abundance of pathogenic bacteria in the three bays (genus level)


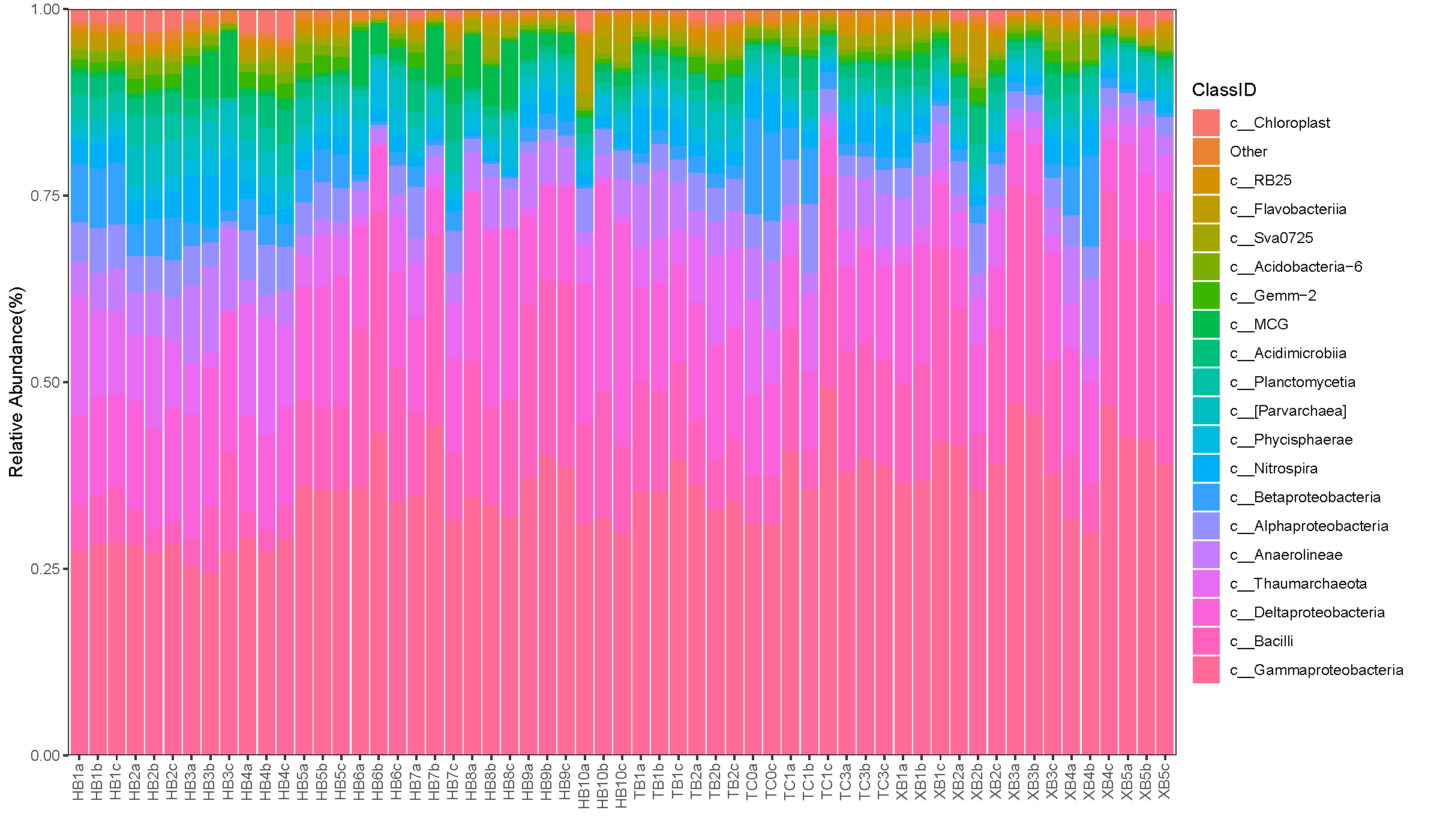


**Supplementary Figure 4**. The composition of microbial community in the three bays (class level, top 20 classes according to relative abundance)

**Supplementary** **Table 1** Seawater qualities and sediment properties in all sampling sites

| Site | Oil  (mg/kg) | TOC^1^  (%) | TN^2^  (mg/kg) | TP^3^  (mg/kg) | Cr  (mg/kg) | As  (mg/kg) | Cu  (mg/kg) | Zn  (mg/kg) | Cd  (mg/kg) | Pb  (mg/kg) | Total Hg  (mg/kg) | T^4^  (℃) | Salinity  (ppt) | pH |
| --- | --- | --- | --- | --- | --- | --- | --- | --- | --- | --- | --- | --- | --- | --- |
| HB1 | 1.8 | 0.106 | 180 | 594 | 24 | 6.36 | 9.5 | 45.7 | 0.056 | 8.8 | 0.008 | 18.2 | 7.2 | 7.95 |
| HB2 | 10.2 | 0.59 | 756 | 605 | 46.8 | 11.8 | 29.2 | 86.3 | 0.112 | 19 | 0.068 | 16.5 | 11.7 | 7.91 |
| HB3 | 3.1 | 0.464 | 664 | 623 | 50.3 | 11.3 | 31.9 | 81.1 | 0.191 | 23.4 | 0.036 | 17.4 | 12.2 | 7.96 |
| HB4 | 2.6 | 0.42 | 295 | 530 | 30 | 10.1 | 12 | 51.6 | 0.082 | 16.4 | 0.035 | 16.1 | 12.4 | 8.04 |
| HB5 | 6.3 | 0.632 | 861 | 631 | 60 | 13.5 | 39.3 | 110 | 0.122 | 27 | 0.053 | 16.2 | 13.4 | 7.91 |
| HB6 | 1.1 | 0.599 | 676 | 585 | 46.2 | 13.3 | 29.3 | 79.4 | 0.112 | 15.8 | 0.064 | 15.8 | 17.1 | 7.94 |
| HB7 | 6.6 | 0.105 | 466 | 542 | 38.1 | 6.67 | 19.1 | 65.2 | 0.07 | 18 | 0.03 | 16.6 | 19 | 7.94 |
| HB8 | 3.6 | 0.368 | 826 | 553 | 45.7 | 8.11 | 24.1 | 77.4 | 0.113 | 22.5 | 0.043 | 15.8 | 30.6 | 8.15 |
| HB9 | 7.3 | 0.478 | 602 | 581 | 43.8 | 10.3 | 28.2 | 79.7 | 0 | 18.8 | 0.156 | 19.9 | 17.8 | 7.89 |
| HB10 | 2.8 | 0.448 | 357 | 459 | 26.8 | 4.18 | 7.3 | 45.4 | 0 | 11.6 | 0.02 | 17.4 | 29 | 8.14 |
| TB1 | 11.8 | 0.553 | 790 | 613 | 55.2 | 12.9 | 31.2 | 90.4 | 0.137 | 28 | 0.056 | 16.3 | 27.6 | 7.99 |
| TB2 | 6.1 | 0.824 | 696 | 582 | 55.4 | 11.2 | 29 | 93.1 | 0.113 | 29.3 | 0.068 | 15.7 | 28.3 | 8 |
| TC0 | 35.5 | 0.372 | 658 | 607 | 60.7 | 14.9 | 38.4 | 110 | 0.14 | 32.6 | 0.067 | 17.9 | 8.7 | 7.75 |
| TC1 | 15.6 | 0.067 | 909 | 657 | 49.6 | 12.8 | 30.8 | 90.2 | 0 | 21.6 | 0.053 | 17.8 | 10.2 | 7.71 |
| TC3 | 10.1 | 0.021 | 717 | 602 | 50.2 | 11.2 | 27.6 | 89.1 | 0.046 | 23.6 | 0.05 | 17.1 | 21.2 | 7.79 |
| XB1 | 10.9 | 0.656 | 863 | 634 | 49.5 | 9.68 | 41 | 98.7 | 0.249 | 22.2 | 0.109 | 17 | 24.8 | 7.99 |
| XB2 | 5.3 | 0.577 | 816 | 579 | 62.1 | 14.8 | 42.1 | 120 | 0.136 | 28.5 | 0.073 | 14.7 | 26 | 8.03 |
| XB3 | 3.8 | 0.668 | 752 | 597 | 53.6 | 12.1 | 33.7 | 101 | 0.139 | 35.7 | 0.057 | 13.9 | 27.1 | 8.01 |
| XB4 | 4 | 0.588 | 699 | 594 | 51.2 | 13.8 | 33.5 | 103 | 0.09 | 22.6 | 0.05 | 14.8 | 27.5 | 8.02 |
| XB5 | 11 | 0.786 | 1060 | 562 | 49 | 10.5 | 29.2 | 87.5 | 0.042 | 20.4 | 0.045 | 14.4 | 30.8 | 8.28 |

1 TOC, Total organic carbon; 2 TN, Total nitrogen; 3 TP, Total phosphorus; 4 T, Temperature

**Supplementary** **Table 2** Alpha diversity of all samples

| Site | No. of reads | Observed OTUs | Goods Coverage | Pielou | Shannon |
| --- | --- | --- | --- | --- | --- |
| HB1a | 77631 | 9058 | 0.90 | 0.78 | 10.27 |
| HB1b | 74796 | 8587 | 0.91 | 0.79 | 10.31 |
| HB1c | 77073 | 8565 | 0.91 | 0.79 | 10.27 |
| HB2a | 72117 | 9500 | 0.90 | 0.82 | 10.82 |
| HB2b | 74802 | 9995 | 0.88 | 0.81 | 10.82 |
| HB2c | 73791 | 9479 | 0.89 | 0.83 | 10.92 |
| HB3a | 72195 | 9492 | 0.89 | 0.82 | 10.82 |
| HB3b | 82219 | 7187 | 0.93 | 0.78 | 9.95 |
| HB3c | 77798 | 5558 | 0.94 | 0.69 | 8.61 |
| HB4a | 76493 | 9757 | 0.89 | 0.80 | 10.56 |
| HB4b | 72192 | 9424 | 0.89 | 0.80 | 10.54 |
| HB4c | 73628 | 10389 | 0.88 | 0.80 | 10.73 |
| HB5a | 74901 | 8433 | 0.90 | 0.76 | 9.97 |
| HB5b | 79682 | 9053 | 0.89 | 0.77 | 10.18 |
| HB5c | 72716 | 8213 | 0.90 | 0.77 | 9.96 |
| HB6a | 77314 | 6684 | 0.92 | 0.66 | 8.35 |
| HB6b | 75545 | 4980 | 0.94 | 0.56 | 6.85 |
| HB6c | 71112 | 8388 | 0.90 | 0.71 | 9.31 |
| HB7a | 72390 | 9220 | 0.89 | 0.77 | 10.08 |
| HB7b | 78700 | 5944 | 0.93 | 0.60 | 7.55 |
| HB7c | 72676 | 9959 | 0.88 | 0.78 | 10.40 |
| HB8a | 78319 | 7277 | 0.91 | 0.68 | 8.66 |
| HB8b | 80684 | 7887 | 0.90 | 0.72 | 9.33 |
| HB8c | 73569 | 9005 | 0.89 | 0.74 | 9.73 |
| HB9a | 76761 | 7082 | 0.91 | 0.65 | 8.34 |
| HB9b | 79691 | 6193 | 0.93 | 0.63 | 7.98 |
| HB9c | 78539 | 6014 | 0.93 | 0.62 | 7.80 |
| HB10a | 71555 | 8914 | 0.89 | 0.74 | 9.65 |
| HB10b | 76302 | 8327 | 0.90 | 0.71 | 9.23 |
| HB10c | 76354 | 8390 | 0.90 | 0.73 | 9.56 |
| TC0a | 81354 | 9094 | 0.89 | 0.71 | 10.16 |
| TC0b | 72349 | 1454 | 0.99 | 0.74 | 5.98 |
| TC0c | 74071 | 8429 | 0.90 | 0.74 | 10.23 |
| TC1a | 55589 | 8035 | 0.90 | 0.79 | 9.35 |
| TC1b | 65226 | 9131 | 0.89 | 0.79 | 10.28 |
| TC1c | 68484 | 6155 | 0.92 | 0.78 | 7.23 |
| TC3a | 72554 | 8861 | 0.89 | 0.77 | 9.48 |
| TC3b | 84294 | 8922 | 0.89 | 0.57 | 9.65 |
| TC3c | 66025 | 9202 | 0.89 | 0.78 | 9.86 |
| TB1a | 78349 | 7952 | 0.90 | 0.72 | 9.16 |
| TB1b | 63432 | 9059 | 0.89 | 0.78 | 9.72 |
| TB1c | 78958 | 9498 | 0.88 | 0.57 | 9.84 |
| TB2a | 76361 | 10781 | 0.87 | 0.72 | 10.64 |
| TB2b | 67013 | 10795 | 0.87 | 0.74 | 10.64 |
| TB2c | 49069 | 10315 | 0.87 | 0.75 | 10.39 |
| XB1a | 72368 | 9024 | 0.89 | 0.75 | 9.81 |
| XB1b | 77786 | 8923 | 0.89 | 0.73 | 9.55 |
| XB1c | 74354 | 7334 | 0.91 | 0.62 | 7.99 |
| XB2a | 79687 | 8269 | 0.90 | 0.69 | 9.03 |
| XB2b | 72079 | 9783 | 0.88 | 0.79 | 10.51 |
| XB2c | 77971 | 8895 | 0.89 | 0.72 | 9.40 |
| XB3a | 80830 | 6583 | 0.92 | 0.57 | 7.27 |
| XB3b | 76369 | 6881 | 0.91 | 0.58 | 7.42 |
| XB3c | 73517 | 7738 | 0.91 | 0.72 | 9.29 |
| XB4a | 67651 | 11823 | 0.85 | 0.81 | 10.93 |
| XB4b | 66803 | 10621 | 0.87 | 0.81 | 10.78 |
| XB4c | 81994 | 6872 | 0.92 | 0.58 | 7.35 |
| XB5a | 66200 | 7387 | 0.91 | 0.61 | 7.84 |
| XB5b | 79551 | 8203 | 0.89 | 0.61 | 7.89 |
| XB5c | 82645 | 8949 | 0.89 | 0.68 | 8.90 |

**Supplementary** **Table 3** The classification of piARGs based on KEGG and CARD Database.

| KO | Gene | Resistance Mechanism | Resistance Class |
| --- | --- | --- | --- |
| K03395 | *aac3-I* | antibiotic inactivation | Aminoglycoside resistance genes (ALR) |
| K00662 | *aacC* | antibiotic inactivation |  |
| K00663 | *aacA* | antibiotic inactivation |  |
| K00984 | *aadA* | antibiotic inactivation |  |
| K05593 | *aadK* | antibiotic inactivation |  |
| K00897 | *aphA* | antibiotic inactivation |  |
| K10673 | *strA* | antibiotic inactivation |  |
| K04343 | *strB* | antibiotic inactivation |  |
| K00561 | *ermC* | antibiotic target alteration | Macrolide resistance genes (MLR) |
| K15632 | *cfr* | antibiotic target alteration |  |
| K08217 | *mef* | antibiotic efflux pump |  |
| K08160 | *mdfA* | antibiotic efflux pump |  |
| K06880 | *ereA_B* | antibiotic inactivation |  |
| K02545 | *mecA* | antibiotic target replacement | Beta-Lactam resistance genes (BLR) |
| K02546 | *mecI* | antibiotic target replacement |  |
| K02547 | *mecR1* | antibiotic target replacement |  |
| K02171 | *blaI* | antibiotic inactivation |  |
| K02172 | *blaR1* | antibiotic inactivation |  |
| K07644 | *cusS* | unclassified |  |
| K07665 | *cusR* | unclassified |  |
| K07260 | *vanY* | antibiotic target alteration | Vancomycin resistance genes (VCR) |
| K15739 | *vanB* | antibiotic target alteration |  |
| K08641 | *vanX* | antibiotic target alteration |  |
| K03367 | *dltA* | unclassified | Cationic antimicrobial peptide (CAMP) resistance genes (CAMPR) |
| K03739 | *dltB* | unclassified |  |
| K03740 | *dltD* | unclassified |  |
| K14188 | *dltC* | unclassified |  |
| K14205 | *mprF* | antibiotic target alteration |  |
| K07637 | *phoQ* | antibiotic target alteration; efflux pump |  |
| K07660 | *phoP* | antibiotic target alteration; efflux pump |  |
| K08477 | *pgtE* | unclassified |  |
| K03585 | *acrA* | antibiotic efflux pump | Multidrug resistance genes (MDR) |
| K12340 | *tolC* | antibiotic efflux pump |  |
| K08170 | *norB* | antibiotic efflux pump |  |
| K13632 | *marA* | antibiotic efflux pump; reduced permeability to antibiotic |  |
| K09476 | *ompF* | reduced permeability to antibiotic |  |
| K08151 | *tetA* | antibiotic efflux pump | Tetracycline resistance genes (TCR) |
| K08168 | *tetB* | antibiotic efflux pump |  |
| K00638 | *catB* | antibiotic inactivation | Phenicol resistance genes (PNR) |
| K08167 | *qacA* | antibiotic efflux pump | Quinolone resistance genes (QNR) |

| Type | Node | Functional Groups |
| --- | --- | --- |
| Carbon Cycle | C1 | Methanotrophy |
|  | C2 | Acetoclastic methanogenesis |
|  | C3 | Methanogenesis by disproportionation of methyl groups |
|  | C4 | Methanogenesis using formate |
|  | C5 | Methanogenesis by CO_2_ reduction with H_2_ |
|  | C6 | Methanogenesis by reduction of methyl compounds with H_2_ |
|  | C7 | Hydrogenotrophic methanogenesis |
|  | C8 | Methanogenesis |
|  | C9 | Methanol oxidation |
|  | C10 | Methylotrophy |
| Nitrogen Cycle | N1 | Aerobic ammonia oxidation |
|  | N2 | Aerobic nitrite oxidation |
|  | N3 | Nitrification |
|  | N4 | Anammox |
|  | N5 | Nitrate denitrification |
|  | N6 | Nitrite denitrification |
|  | N7 | Nitrous oxide denitrification |
|  | N8 | Denitrification |
|  | N9 | Nitrogen fixation |
|  | N10 | Nitrate ammonification |
|  | N11 | Nitrite ammonification |
|  | N12 | Nitrite respiration |
|  | N13 | Nitrate respiration |
|  | N14 | Nitrate reduction |
|  | N15 | Nitrogen respiration |
| Sulfur  Cycle | S1 | Sulfate respiration |
|  | S2 | Sulfur respiration |
|  | S3 | Dark sulfite oxidation |
|  | S4 | Sulfite respiration |
|  | S5 | Thiosulfate respiration |
|  | S6 | Respiration of sulfur compounds |
|  | S7 | Dark sulfide oxidation |
|  | S8 | Dark sulfur oxidation |
|  | S9 | Dark thiosulfate oxidation |
|  | S10 | Dark oxidation of sulfur compounds |
| Degradation | D1 | Aromatic hydrocarbon degradation |
|  | D2 | Aromatic compound degradation |
|  | D3 | Aliphatic non-methane hydrocarbon degradation |
|  | D4 | Hydrocarbon degradation |
|  | D5 | Plastic degradation |

**Supplementary** **Table 4** Functional groups related to C, N, S cycles and degradation metabolisms

| Network | Nodes | Edges | Average degree | Modularity | Positive correlation | Negative correlation |
| --- | --- | --- | --- | --- | --- | --- |
| gHB | 160 | 406 | 5.075 | 0.582 | 62.32% | 37.68% |
| gTB | 154 | 338 | 4.39 | 0.675 | 56.8% | 43.2% |
| gXB | 117 | 287 | 4.906 | 0.441 | 82.23% | 17.77% |
| fHB | 47 | 109 | 4.638 | 0.505 | 75.23% | 24.77% |
| fTB | 57 | 93 | 3.263 | 0.58 | 61.29% | 38.71% |
| fXB | 43 | 137 | 6.372 | 0.176 | 87.59% | 12.41% |

**Supplementary** **Table 5** Properties of networks

**Supplementary** **Table 6** Microbial genus with higher degree (>average degree) in the different networks

| Network | Node | Degree | Microbial genus |
| --- | --- | --- | --- |
| gHB | G1491 | 15 | k_Bacteria;p_Proteobacteria;c_Gammaproteobacteria;o_Pseudomonadales;f_Pseudomonadaceae;Other |
|  | G659 | 15 | k_Bacteria;p_Firmicutes;c_Bacilli;o_Lactobacillales;f_Enterococcaceae;g_Enterococcus |
|  | G1479 | 14 | k_Bacteria;p_Proteobacteria;c_Gammaproteobacteria;o_Pseudomonadales;f_Moraxellaceae;g_Acinetobacter |
|  | G1406 | 13 | k_Bacteria;p_Proteobacteria;c_Gammaproteobacteria;o_Enterobacteriales;f_Enterobacteriaceae;g_Citrobacter |
|  | G618 | 13 | k_Bacteria;p_Firmicutes;c_Bacilli;o_Bacillales;f_[Exiguobacteraceae];g_ |
|  | G624 | 13 | k_Bacteria;p_Firmicutes;c_Bacilli;o_Bacillales;f_Bacillaceae;g_Bacillus |
|  | G1490 | 11 | k_Bacteria;p_Proteobacteria;c_Gammaproteobacteria;o_Pseudomonadales;f_Pseudomonadaceae;g_Pseudomonas |
|  | G565 | 11 | k_Bacteria;p_Cyanobacteria;c_Chloroplast;o_Stramenopiles;f_;g_ |
|  | G1353 | 10 | k_Bacteria;p_Proteobacteria;c_Gammaproteobacteria;o_Alteromonadales;f_125ds10;g_ |
|  | G1416 | 10 | k_Bacteria;p_Proteobacteria;c_Gammaproteobacteria;o_Enterobacteriales;f_Enterobacteriaceae;Other |
|  | G1643 | 10 | k_Bacteria;p_Verrucomicrobia;c_Opitutae;o_;f_;g_ |
|  | G847 | 10 | k_Bacteria;p_Nitrospirae;c_Nitrospira;o_Nitrospirales;f_Nitrospiraceae;g_ |
|  | G1124 | 9 | k_Bacteria;p_Proteobacteria;c_Betaproteobacteria;o_Burkholderiales;f_Comamonadaceae;g_Hydrogenophaga |
|  | G651 | 9 | k_Bacteria;p_Firmicutes;c_Bacilli;o_Bacillales;Other;Other |
|  | G293 | 7 | k_Bacteria;p_Bacteroidetes;c_[Saprospirae];o_[Saprospirales];f_Saprospiraceae;g_ |
|  | G491 | 7 | k_Bacteria;p_Chloroflexi;c_Anaerolineae;o_Caldilineales;f_Caldilineaceae;g_ |
| gTB | G667 | 18 | k_Bacteria;p_Firmicutes;c_Bacilli;o_Lactobacillales;f_Streptococcaceae;g_Lactococcus |
|  | G65 | 13 | k_Archaea;p_Euryarchaeota;c_Thermoplasmata;o_E2;f_;g_ |
|  | G1406 | 12 | k_Bacteria;p_Proteobacteria;c_Gammaproteobacteria;o_Enterobacteriales;f_Enterobacteriaceae;g_Citrobacter |
|  | G1479 | 12 | k_Bacteria;p_Proteobacteria;c_Gammaproteobacteria;o_Pseudomonadales;f_Moraxellaceae;g_Acinetobacter |
|  | G1491 | 11 | k_Bacteria;p_Proteobacteria;c_Gammaproteobacteria;o_Pseudomonadales;f_Pseudomonadaceae;Other |
|  | G618 | 11 | k_Bacteria;p_Firmicutes;c_Bacilli;o_Bacillales;f_[Exiguobacteraceae];g_ |
|  | G1478 | 10 | k_Bacteria;p_Proteobacteria;c_Gammaproteobacteria;o_Pseudomonadales;f_Moraxellaceae;g_ |
|  | G1490 | 10 | k_Bacteria;p_Proteobacteria;c_Gammaproteobacteria;o_Pseudomonadales;f_Pseudomonadaceae;g_Pseudomonas |
|  | G1493 | 10 | k_Bacteria;p_Proteobacteria;c_Gammaproteobacteria;o_PYR10d3;f_;g_ |
|  | G624 | 9 | k_Bacteria;p_Firmicutes;c_Bacilli;o_Bacillales;f_Bacillaceae;g_Bacillus |
|  | G24 | 8 | k_Archaea;p_Crenarchaeota;c_Thaumarchaeota;o_Cenarchaeales;f_Cenarchaeaceae;g_Nitrosopumilus |
|  | G619 | 8 | k_Bacteria;p_Firmicutes;c_Bacilli;o_Bacillales;f_[Exiguobacteraceae];g_Exiguobacterium |
|  | G1062 | 7 | k_Bacteria;p_Proteobacteria;c_Alphaproteobacteria;o_Rhodospirillales;f_Rhodospirillaceae;g_ |
|  | G1100 | 7 | k_Bacteria;p_Proteobacteria;c_Betaproteobacteria;o_;f_;g_ |
|  | G1154 | 7 | k_Bacteria;p_Proteobacteria;c_Betaproteobacteria;o_Hydrogenophilales;f_Hydrogenophilaceae;g_Thiobacillus |
|  | G1138 | 5 | k_Bacteria;p_Proteobacteria;c_Betaproteobacteria;o_Burkholderiales;f_Comamonadaceae;Other |
|  | G1297 | 5 | k_Bacteria;p_Proteobacteria;c_Deltaproteobacteria;o_NB1-j;f_NB1-i;g_ |
|  | G1487 | 5 | k_Bacteria;p_Proteobacteria;c_Gammaproteobacteria;o_Pseudomonadales;f_Pseudomonadaceae;g_ |
|  | G651 | 5 | k_Bacteria;p_Firmicutes;c_Bacilli;o_Bacillales;Other;Other |
| gXB | G1479 | 21 | k_Bacteria;p_Proteobacteria;c_Gammaproteobacteria;o_Pseudomonadales;f_Moraxellaceae;g_Acinetobacter |
|  | G618 | 21 | k_Bacteria;p_Firmicutes;c_Bacilli;o_Bacillales;f_[Exiguobacteraceae];g_ |
|  | G659 | 21 | k_Bacteria;p_Firmicutes;c_Bacilli;o_Lactobacillales;f_Enterococcaceae;g_Enterococcus |
|  | G1409 | 20 | k_Bacteria;p_Proteobacteria;c_Gammaproteobacteria;o_Enterobacteriales;f_Enterobacteriaceae;g_Klebsiella |
|  | G1490 | 20 | k_Bacteria;p_Proteobacteria;c_Gammaproteobacteria;o_Pseudomonadales;f_Pseudomonadaceae;g_Pseudomonas |
|  | G624 | 20 | k_Bacteria;p_Firmicutes;c_Bacilli;o_Bacillales;f_Bacillaceae;g_Bacillus |
|  | G668 | 20 | k_Bacteria;p_Firmicutes;c_Bacilli;o_Lactobacillales;f_Streptococcaceae;g_Streptococcus |
|  | G667 | 15 | k_Bacteria;p_Firmicutes;c_Bacilli;o_Lactobacillales;f_Streptococcaceae;g_Lactococcus |
|  | G1406 | 14 | k_Bacteria;p_Proteobacteria;c_Gammaproteobacteria;o_Enterobacteriales;f_Enterobacteriaceae;g_Citrobacter |
|  | G1499 | 12 | k_Bacteria;p_Proteobacteria;c_Gammaproteobacteria;o_Thiotrichales;f_Piscirickettsiaceae;g_Methylophaga |
|  | G1491 | 11 | k_Bacteria;p_Proteobacteria;c_Gammaproteobacteria;o_Pseudomonadales;f_Pseudomonadaceae;Other |
|  | G1271 | 7 | k_Bacteria;p_Proteobacteria;c_Deltaproteobacteria;o_Myxococcales;f_;g_ |

**Supplementary** **Table 7** The relative abundance of ARG profile (copies of ARG per copy of 16S rRNA) based on metagenomics analysis.

| site | 2012S2 | 2012Z1 | 2012Z2 | 2012Z3 | 2012Z4 | 2014Z1 | 2014Z2 | 2014Z3 | 2014Z4 | 2014Z5 |
| --- | --- | --- | --- | --- | --- | --- | --- | --- | --- | --- |
| aminoglycoside | 0.0083 | 0.0059 | 0.0099 | 0.0042 | 0.0116 | 0.0097 | 0.0031 | 0.0059 | 0.0032 | 0.0053 |
| bacitracin | 0.0278 | 0.0234 | 0.0286 | 0.0116 | 0.0165 | 0.0181 | 0.0118 | 0.0107 | 0.0064 | 0.0075 |
| beta-lactam | 0.0028 | 0.0024 | 0.0070 | 0.0027 | 0.0045 | 0.0042 | 0.0020 | 0.0020 | 0.0007 | 0.0022 |
| chloramphenicol | 0.0027 | 0.0036 | 0.0012 | 0.0004 | 0.0018 | 0.0005 | 0.0002 | 0.0005 | 0.0002 | 0.0005 |
| fosfomycin | 0.0044 | 0.0039 | 0.0115 | 0.0028 | 0.0034 | 0.0018 | 0.0008 | 0.0011 | 0.0010 | 0.0018 |
| fosmidomycin | 0.0011 | 0.0009 | 0.0007 | 0.0002 | 0.0005 | 0.0015 | 0.0006 | 0.0005 | 0.0003 | 0.0006 |
| kasugamycin | 0.0003 | 0.0002 | 0.0002 | 0.0012 | 0.0006 | 0.0004 | 0.0001 | 0.0005 | 0.0002 | 0.0005 |
| macrolide-lincosamide-streptogramin | 0.0120 | 0.0095 | 0.0169 | 0.0087 | 0.0165 | 0.0142 | 0.0085 | 0.0102 | 0.0074 | 0.0096 |
| multidrug | 0.0599 | 0.0508 | 0.0579 | 0.0274 | 0.0462 | 0.0451 | 0.0219 | 0.0291 | 0.0211 | 0.0276 |
| polymyxin | 0.0007 | 0.0006 | 0.0008 | 0.0010 | 0.0008 | 0.0014 | 0.0007 | 0.0013 | 0.0006 | 0.0009 |
| puromycin | 0.0000 | 0.0000 | 0.0000 | 0.0000 | 0.0000 | 0.0000 | 0.0000 | 0.0000 | 0.0000 | 0.0000 |
| quinolone | 0.0009 | 0.0014 | 0.0014 | 0.0013 | 0.0015 | 0.0004 | 0.0003 | 0.0005 | 0.0004 | 0.0006 |
| rifamycin | 0.0001 | 0.0004 | 0.0006 | 0.0001 | 0.0004 | 0.0004 | 0.0003 | 0.0002 | 0.0001 | 0.0000 |
| sulfonamide | 0.0077 | 0.0080 | 0.0005 | 0.0001 | 0.0002 | 0.0023 | 0.0011 | 0.0012 | 0.0001 | 0.0000 |
| tetracycline | 0.0088 | 0.0068 | 0.0107 | 0.0070 | 0.0122 | 0.0054 | 0.0016 | 0.0038 | 0.0023 | 0.0050 |
| trimethoprim | 0.0008 | 0.0008 | 0.0002 | 0.0003 | 0.0001 | 0.0000 | 0.0006 | 0.0007 | 0.0000 | 0.0008 |
| unclassified | 0.0118 | 0.0130 | 0.0078 | 0.0159 | 0.0161 | 0.0117 | 0.0148 | 0.0160 | 0.0172 | 0.0153 |
| vancomycin | 0.0079 | 0.0049 | 0.0127 | 0.0068 | 0.0170 | 0.0096 | 0.0027 | 0.0062 | 0.0026 | 0.0055 |
| **total abundance** | **0.1580** | **0.1366** | **0.1684** | **0.0918** | **0.1500** | **0.1267** | **0.0711** | **0.0901** | **0.0640** | **0.0837** |

**Supplementary** **Table 8** The relative abundance of ARG profile (copies of ARG per prokaryotic cell) based on metagenomics analysis.

| site | 2012S2 | 2012Z1 | 2012Z2 | 2012Z3 | 2012Z4 | 2014Z1 | 2014Z2 | 2014Z3 | 2014Z4 | 2014Z5 |
| --- | --- | --- | --- | --- | --- | --- | --- | --- | --- | --- |
| aminoglycoside | 0.0104 | 0.0070 | 0.0173 | 0.0053 | 0.0183 | 0.0103 | 0.0029 | 0.0056 | 0.0030 | 0.0053 |
| bacitracin | 0.0349 | 0.0279 | 0.0498 | 0.0148 | 0.0261 | 0.0191 | 0.0110 | 0.0102 | 0.0060 | 0.0076 |
| beta-lactam | 0.0035 | 0.0028 | 0.0121 | 0.0034 | 0.0071 | 0.0045 | 0.0019 | 0.0019 | 0.0007 | 0.0022 |
| chloramphenicol | 0.0034 | 0.0042 | 0.0020 | 0.0006 | 0.0029 | 0.0006 | 0.0002 | 0.0005 | 0.0002 | 0.0005 |
| fosfomycin | 0.0055 | 0.0047 | 0.0200 | 0.0036 | 0.0054 | 0.0019 | 0.0008 | 0.0010 | 0.0010 | 0.0018 |
| fosmidomycin | 0.0013 | 0.0010 | 0.0012 | 0.0002 | 0.0008 | 0.0016 | 0.0006 | 0.0005 | 0.0003 | 0.0006 |
| kasugamycin | 0.0004 | 0.0002 | 0.0003 | 0.0016 | 0.0010 | 0.0004 | 0.0001 | 0.0005 | 0.0002 | 0.0005 |
| macrolide-lincosamide-streptogramin | 0.0151 | 0.0114 | 0.0295 | 0.0112 | 0.0260 | 0.0150 | 0.0079 | 0.0097 | 0.0069 | 0.0096 |
| multidrug | 0.0750 | 0.0605 | 0.1010 | 0.0350 | 0.0730 | 0.0477 | 0.0204 | 0.0278 | 0.0197 | 0.0277 |
| polymyxin | 0.0009 | 0.0007 | 0.0013 | 0.0013 | 0.0013 | 0.0015 | 0.0007 | 0.0012 | 0.0005 | 0.0009 |
| puromycin | 0.0000 | 0.0000 | 0.0000 | 0.0000 | 0.0000 | 0.0000 | 0.0000 | 0.0000 | 0.0000 | 0.0000 |
| quinolone | 0.0012 | 0.0017 | 0.0025 | 0.0017 | 0.0023 | 0.0004 | 0.0003 | 0.0005 | 0.0004 | 0.0006 |
| rifamycin | 0.0002 | 0.0005 | 0.0011 | 0.0001 | 0.0006 | 0.0004 | 0.0003 | 0.0002 | 0.0001 | 0.0000 |
| sulfonamide | 0.0096 | 0.0095 | 0.0008 | 0.0002 | 0.0003 | 0.0024 | 0.0010 | 0.0011 | 0.0001 | 0.0000 |
| tetracycline | 0.0110 | 0.0081 | 0.0187 | 0.0089 | 0.0193 | 0.0057 | 0.0015 | 0.0036 | 0.0021 | 0.0050 |
| trimethoprim | 0.0010 | 0.0010 | 0.0004 | 0.0004 | 0.0001 | 0.0000 | 0.0006 | 0.0007 | 0.0000 | 0.0008 |
| unclassified | 0.0147 | 0.0155 | 0.0136 | 0.0203 | 0.0254 | 0.0123 | 0.0137 | 0.0153 | 0.0160 | 0.0153 |
| vancomycin | 0.0099 | 0.0059 | 0.0221 | 0.0087 | 0.0269 | 0.0102 | 0.0025 | 0.0059 | 0.0025 | 0.0055 |
| **total abundance** | **0.1979** | **0.1627** | **0.2940** | **0.1173** | **0.2370** | **0.1340** | **0.0663** | **0.0862** | **0.0597** | **0.0838** |

**Supplementary** **Table 9** Abundance-weighted average *rrn* copy number of microbial communities in the previous study

| site | Abundance-weighted average *rrn* copy number |
| --- | --- |
| 2012S2 | 6.453 |
| 2012Z1 | 5.009 |
| 2012Z2 | 5.704 |
| 2012Z3 | 6.456 |
| 2012Z4 | 6.875 |
| 2014Z1 | 3.278 |
| 2014Z2 | 2.962 |
| 2014Z3 | 3.416 |
| 2014Z4 | 2.953 |
| 2014Z5 | 3.037 |
